# Dementia in long-term Parkinson’s disease patients: a multicentre retrospective study

**DOI:** 10.1038/s41531-019-0106-4

**Published:** 2020-01-07

**Authors:** Jennifer Y. Y. Szeto, Courtney C. Walton, Alexandra Rizos, Pablo Martinez-Martin, Glenda M. Halliday, Sharon L. Naismith, K. Ray Chaudhuri, Simon J. G. Lewis

**Affiliations:** 1 Parkinson’s Disease Research Clinic, Brain and Mind Centre and Central Clinical School, Faculty of Medicine and Health, University of Sydney, Sydney, NSW Australia; 2 Parkinson Foundation International Centre of Excellence, King’s College Hospital and Kings College London, London, UK; 3 National Institute for Health Research (NIHR) Mental Health Biomedical Research Centre and Dementia Unit at South London and Maudsley NHS Foundation Trust and King’s College London, London, UK; 4 Department of Basic and Clinical Neuroscience, The Maurice Wohl Clinical Neuroscience Institute, Kings College London, London, UK; 5 Centre for Networked Biomedical Research on Neurodegenerative Diseases (CIBERNED), Carlos III Institute of Health, Madrid, Spain; 6 Brain and Mind Centre and Central Clinical School, Faculty of Medicine and Health, University of Sydney, Sydney, NSW Australia; 7 Healthy Brain Ageing Program, School of Psychology; Brain and Mind Centre & The Charles Perkins Centre, University of Sydney, Sydney, NSW Australia

**Keywords:** Dementia, Parkinson's disease

## Abstract

While several studies have investigated the clinical progression of cognitive decline in Parkinson’s disease (PD) patients, there has been a paucity of data on specifically evaluating PD patients with a disease duration of over 20 years. This study retrospectively investigated the frequency of dementia in PD (PDD) patients with a disease duration of over 20 years assessed in research clinics across the UK and Australia. Data from 2327 PD patients meeting the United Kingdom Parkinson’s Disease Society Brain Bank Criteria was pooled. A diagnosis of probable PDD was made according to the Movement Disorder Society Level 1 criteria. Thirty-six participants had a disease duration of at least 20 years. Of the 36 patients, only 7 (19%) were classified as probable PDD. Compared to PD patients without dementia, those with dementia had lower levels of educational attainment and exhibited more severe motor features. Additionally, 34 out of the 36 patients (94%) exhibited a non-tremor dominant phenotype. No significant differences in age, age onset, disease duration, dopaminergic medication use, and sex distribution were observed between PD patients with and without dementia. Findings from the present study suggest that the prevalence of dementia in long-term PD patients may be lower than anticipated and suggest that the trajectory of cognitive decline in PD patients can be different. These findings highlight the need to investigate factors that might affect the outcome of cognitive decline in long-term PD patients, which may lead to the determination of potential modulating factors in the development of dementia in these patients.

## Introduction

Parkinson’s disease (PD) is a progressive neurodegenerative disorder with a wide variety of clinical symptoms. In addition to its classic motor features (i.e. tremor, rigidity, bradykinesia, and postural instability), non-motor symptoms (e.g. cognitive impairment, sleep disturbances, depression, and hallucinations) are now widely accepted as part of the clinical spectrum.^1,2^ In particular, the presence of dementia in PD (PDD) represents one of the most significant non-motor symptoms, especially in more advanced disease.^3,4^ While the prevalence has varied across studies depending on the diagnostic criteria employed and the nature of the study population,^5^ a previous systematic review has reported the point-prevalence of PDD to be approximately 30%.^6^ Significantly, such cognitive decline is associated with increased mortality, impairments in well-being, caregiver strain as well as increased healthcare and institutionalisation costs.^7–9^ Therefore, the risk of developing dementia in PD is often an important topic for patients and their families given its significant impact.

The Sydney Multicentre Study represented the first comprehensive natural history study to follow a single cohort of newly diagnosed PD patients (with an average onset at around 60 years of age) over the subsequent 20 years of their disease.^10^ On the basis of data from those patients assessed at 5, 10, 15 and 20- years^10–13^ the authors reported that by the time of their final review (around 80 years of age), 25 of the 30 remaining patients (83%) from the original study had developed dementia.^10^ A second analysis of the same cohort at 20 years of disease revealed that cognitive decline began at a similar chronological age (70–75 years) in most patients regardless of when their PD started.^14^ These and similar findings from other longitudinal studies^15,16^ have raised the question as to whether dementia is inevitable in individuals with long-term idiopathic PD.

While several studies have investigated the clinical progression of cognitive decline in PD patients, there has been a paucity of data on specifically evaluating PD patients with a disease duration of over 20 years.^17^ The current study retrospectively investigated the frequency of dementia in PD patients with a disease duration of over 20 years assessed in research clinics across the UK and Australia. Our aim was to determine whether these patients would demonstrate the high rates of dementia that might be anticipated from the previous longitudinal cohort studies, and if there were any phenotypic features that might ameliorate against this transition.

## Results

The study identified 36 participants who had a disease duration of at least 20 years at the time of assessment, representing 1.5% of all the participants pooled across the research clinics in the UK and Australia (Table 1). With regard to the Australian cohort, 11 of the 18 participants were enrolled into the brain donation programme, and home visits were carried out for 8 participants. Of the 36 participants with at least 20 years of disease duration, only 7 patients (19%) were classified as probable PDD (1 from the UK cohort and 6 from the Australian cohort). Table 1 demonstrates the proportion and clinical features of PD patients with and without dementia as defined by the MDS PDD Level 1 Criteria.

**Table 1:** Mean (SD) scores for demographic and clinical features of PD patients with disease duration of ≥20 years with and without dementia

	Mean (SD)			
	Total	PDwD	PDD	PDwD vs. PDD
Total number (%)	36 (100%)	29 (81%)	7 (19%)	N/A
Age, years	72.64 (8.6)	71.59 (7.8)	77.23 (11.0)	*F* = 2.51, *p* = 0.12
Age onset, years	49.65 (8.9)	48.37 (8.0)	53.84 (10.9)	*F* = 2.28, *p* = 0.14
Disease duration	22.99 (4.6)	23.23 (4.2)	23.39 (4.1)	*U* = 85.50, *p* = 0.53
H & Y, range (median)	1–5 (3)	1–5 (3)	3–5(4)	*U* = 33.00, *p* = 0.004
Gender, male	21/36	17/29	4/7	*X* = 0.005, *p* = 1
Education, years	14.47 (4.3)	15.41 (4.1)	10.00 (2.2)	*U* = 30.00, *p* = 0.002
DDE, mg	687.65 (545.3)	726.44 (538.85)	1158.39 (833.4)	*U* = 70.50, *p* = 0.22
MMSE, Total	25 (5.7)	27.28 (3.7)	18.14 (7.1)	*U* = 16.50, *p* < 0.001
**Sub-analysis of motor phenotype score**				
Australian cohort				
Total number (%)	18 (100%)	12 (67%)	6 (33%)	N/A
Motor phenotype score	0.23 (0.2)	0.16 (0.1)	0.37 (0.2)	*F* = 5.56, *p* = 0.031
Tremor score	0.51 (0.5)	0.34 (0.3)	0.85 (0.6)	*F* = 6.10, *p* = 0.025
Non-tremor score	2.08 (0.7)	1.96 (0.7)	2.32 (0.8)	*F* = 1.00, *p* = 0.33

Compared with PD patients without dementia (PDwD), PDD patients had a lower level of educational attainment. The groups were similar in years of disease durations, sex distribution, and dopaminergic medication use. Other key phenotypic features are described below.

### Age and age onset

Of the seven participants characterised as PDD, their ages at assessment ranged from 58 to 91, with 6/7 of them over the age of 70. For the remaining 29 PDwD patients, the age range was between 59 and 95, with 19 of them over the age of 70. The frequency of PDD was similar between patients of older and younger than 70 years (*χ*^2^ = 1.08, *p* = 0.40). Specifically, there were 19/29 PDwD patients versus 6/7 PDD patients who were over the age of 70 years and 10/29 PDwD patients versus 1/7 PDD patients under 70 years.

No significant difference in age of onset was observed between PD patients with and without dementia. A total of 16 participants had a disease onset younger than 50 years (range = 30–49.56 years), with the remaining 20 developing PD between the age of 50 and 67 years. Furthermore, the frequency of PDD was similar between patients with disease onset age above and below the age of 50 years (*χ*^2^ = −0.89, *p* = 0.43).

### Severity of motor symptoms

Compared to those without dementia, PDD patients had more severe motor features with a greater impairment in balance. Of the 36 participants, 13 were characterised H & Y stage IV and V, whereas the others were between stages I–III. In particular, of the seven PDD participants, six were characterised by having H & Y stages IV and V.

### Motor phenotype

Of the 36 patients in the present study, 34 (94%) exhibited a non-tremor dominant (TD) phenotype. Specifically, all the 18 PD patients from the Australian group had a non-TD phenotype (range 0.03–0.70). Similarly, of the 18 PD patients from the UK cohort, only 1 patient exhibited a TD phenotype (motor phenotype score = 1.50) and 1 exhibited a mixed phenotype (motor phenotype score = 1) with the remaining patients again exhibiting a non-TD phenotype (range = 0.05–0.75).

Sub-analysis of the patients from the Australian cohort where a MDS-UPDRS assessment was available revealed a significant difference in the motor phenotype score between those patients with and without dementia. An investigation of difference in motor phenotype score was not performed between PD patients with and without dementia in the UK cohort, as only one person was characterised as PDD. The Australian PDD group demonstrated a higher motor phenotype score than those in the PDwD group. Closer examinations of the motor phenotype scores, which were derived from tremor/non-tremor ratios, suggested that scores on both tremor and non-tremor items were higher in the PDD group than in the PDwD group (TD/NTD ratio = 0.85/2.32 in the PDD group versus 0.34/1.96 in the PDwD group). However, this difference was not statistically significant.

### Family history of PD

Additional information on family history of PD was obtained from the Australian cohort. Of the 18 patients, 4 patients (22%) reported a family history of PD, of which 3 were characterised as PDwD and only 1 had developed PDD.

## Discussion

To date, only a limited number of studies have investigated the clinical features of PD patients with disease duration of 20 years or more, as this is a small number of people with PD.^10,17^ We identified 36 such patients from a cohort of 2327 patients (1.5%) and investigated the frequency of dementia within them using data from assessments ≥20 years from diagnosis. Using the MDS Level 1 PDD criteria, only 7 of the 36 (19%) participants met criteria for PDD after 20 years. This finding is in contradiction from what might have been anticipated from previous studies in long-term PD (e.g. 83% in the Sydney Multicentre Study^10^) and suggests that the trajectory of cognitive decline in PD patients can be different, warranting the identification of factors that might affect the trajectory of PDD.

While findings on the effect of age onset and disease duration on the presentation of PDD are more controversial,^14,18–21^ older chronological age has been consistently reported in previous research to be an independent and key risk factor for PDD.^10,21,22^ In contrast to previous findings of the impact of chronological age on the development of PDD, we did not observe significant differences in age between our PDD and PDwD groups. Of note, disease duration and onset age were also both similar between the two groups. Indeed, a more advanced age was detected in the PDD group as compared to the PDwD group, yet, the small sample size in the PDD group could have potentially undermined statistically significant difference in chronological age between the two groups. Alternatively, it is also possible that age exerts additive or synergistic effects in contributing to the development of PDD or phenotypic expression in patients in the present study, rather than playing an independent role.^23–25^ In a study by Levy et al.^25^, the separate and combined effects of age and severity of extrapyramidal signs (EPSs; which have found to be consistently associated with incident dementia in PD in longitudinal studies) on the risk of developing PDD were investigated. Results from the study suggest that rather than exerting separate effects, age and severity of EPSs exerted combined effects on the risk of developing PDD.^25^ Therefore, while there has been a discussion on the role of individual risk factors for the development of dementia in PD, perhaps more focus needs to be placed on how the combined effect of risk factors impacts upon the trajectory of cognitive decline in long-term PD.

One potential explanation for the discrepancy in PDD prevalence between the present study and that of previous prospective studies relates to the less severe motor symptoms of the entire sample. For example, in the Sydney Multicentre Study the mean H & Y stage was 4.2 at 20 years as compared to that observed in the present study where the mean H & Y stage was 3.19. Indeed, in the present study motor impairment was also found to be more severe in the PDD group as compared to the PDwD group and nearly all of the PDD patients (six out of seven) had late-stage disease (H & Y IV–V). The finding that dementia occurred more frequently in patients with advanced motor impairment is consistent with previous work.^3^ This perhaps reflects more profound neuropathological lesions in PDD patients, which is likely to involve nigral and extra-nigral pathology.^26–29^ While severe dopamine depletion in the striatum due to loss of dopaminergic neurons in the nigrostriatal pathway is well recognised as the predominant histological feature of PD, it has also been implicated in the regulation of corticostriatal pathways and functional connectivity that is important in cognitive processes.^26,30,31^ As such, the more profound dopamine dysfunction is likely to have impact upon the functional connection between regions involved in cognitive processes. Newer concepts also suggest the non-motor endophenotypes of PD where either Park-Cog subtype (mainly with early mild cognitive impairment or apathy) or cholinergic phenotype is thought to exist.^2,32^ It is possible that the original studies included these subtypes which have a higher risk of progression to dementia.^33^ Nevertheless, it remains unclear why 7 out of the 13 of PD patients with late-stage disease remained dementia-free. It is likely that other progressive pathologies seen in PD (e.g. dopaminergic, noradrenergic, serotonergic, and cholinergic pathologies) exert synergistic effects, which lead to the clinical manifestation of motor and cognitive impairment once individual thresholds are crossed.^3,26,34^ For example, while the emergence of motor symptoms in PD often occurs against the background of severe dopamine deficits, the involvement of the non-dopaminergic pathologies system such as the cholinergic system has also been implicated in contributing to worsening motor symptoms in PD (e.g. refs. ^35,36^). In parallel, previous neuropathological and neuroimaging studies have suggested the degeneration of the cholinergic system to play a major role in the aetiology of non-tremor motor features in PD including postural instability and gait.^26,35,37^ Importantly, the involvement of the cholinergic system has also been discussed extensively in cognitive dysfunction and dementia in PD.^26,37–39^ Previous research reported that apart from more substantial dopaminergic deficits, PDD patients also exhibited additional cholinergic deficits compared with non-demented PD patients.^39^ Taken together, considering the role of altered cholinergic neurotransmission on motor and cognitive functioning in PD, the more severe level of motor impairment and TD/NTD ratio observed in the PDD group may reflect a more widespread pathology that includes additional cholinergic denervation.

Another potential explanation for the discrepancy in PDD prevalence between the present study and that of the Sydney Multicentre Study relates to the employment of different criteria to define PDD. While a more extensive neuropsychological test battery was employed to determine cognitive impairment in the Sydney Multicentre Study, PDD was defined based on the utilisation of MMSE/MoCA in the present study.

Interestingly, findings from this study revealed that only 1 out of the 36 participants exhibited a TD phenotype, which might be less than would be anticipated from previous studies suggesting that such patients are conferred with a more benign course and slower disease progression^15,17,40,41^. While the overrepresentation of patients with non-TD feature may reflect an artefact of how the motor phenotypes were classified in the present study, such overrepresentation more likely suggests the transition of predominant motor features over the long disease course. Previous research has documented that motor phenotypes in PD can change over the course of the disease, with the majority of those transitioning from TD to NTD features later in the disease course.^42–45^ Importantly, such transition could be strongly associated with the development of cognitive decline.^43–45^ In a population-based observational study of 171 non-demented PD patients,^44^ the association between changes in predominant motor symptoms and the development of dementia was investigated over 8 years. This study found that the transition from TD to NTD was unidirectional and irreversible with 44% of participants transitioning from a TD phenotype to that of NTD over 8 years. This transition was due to the development of more severe NTD features in addition to pre-existing tremor features. Importantly, such a transition has been found to be strongly associated with accelerated cognitive decline and the subsequent development of dementia. Of those who transitioned into an NTD phenotype, approximately 50% became demented within the following 4 years, whereas none of the patients with persistent TD features became demented at follow-up. As information on the presentation of initial motor symptoms was not captured in the present study, it was impossible to determine the stability of motor phenotypes in the long-term PD studied here as well as the extent to which the initial presence of tremor features is “protected” against future cognitive decline as had been suggested in previous research.^17,44^ Future longitudinal studies will be crucial in exploring the transition of motor phenotypes in PD and factors that mediate such transition, as well as how such transition is associated with the development of PDD.

In addition to the overrepresentation of PD patients with NTD features, the sub-analysis of motor phenotype scores within the Australian cohort has revealed that while all participants exhibited an NTD phenotype, individuals with PDD exhibited a higher TD/NTD ratio as compared to those with PDwD. A close examination regarding the TD and NTD scores showed that although not significantly different between the groups, individuals in the PDD group exhibited a more severe level of both tremor and non-tremor features as compared to those in the PDwD group. These findings reinforce the viewpoint that there is likely to be a more profound neuropathology associated with PDD.

As demonstrated by findings in the present study, individuals in the PDD group had a lower level of educational attainment than those in the PDwD group. While a number of studies have documented a higher risk of developing dementia in PD patients with lower levels of education, only a limited number of studies have explored the mechanisms underlying such an association. Lucero et al.^46^ investigated whether or not the correlation between cortical β-amyloid accumulation (which has been documented to occur in more than half of PDD patients^47^) and clinical expression of cognitive impairment is modified by educational attainment in PD patients. They found that in those with lower level of education, higher cortical β-amyloid correlated with worse cognitive function. Conversely, among those with a higher level of education, higher cortical β-amyloid did not correlate with worse cognitive function, despite demonstrating similar levels of pathology to that observed in the lower education group. It is possible that educational attainment, which is generally viewed as a proxy for cognitive reserve, may exert a compensatory effect by mitigating the association between pathological burden and cognitive decline (i.e. increasing an individual’s ability to withstand a burden of neurodegenerative brain disease before expressing clinical dementia).^46,48,49^ However, such hypotheses will have to be confirmed in future studies. Understanding how education mitigates such associations represents a significant gap in PD research and could have important treatment implications for developing cognitive training strategies in PD patients looking to delay the onset of cognitive decline associated with their disease.^46,50^ The currently available evidence suggests that cognitive training programmes are efficacious on short-term neuropsychological measures in PD populations,^51^ but longitudinal data are needed.

Several methodological problems of the current study should be noted. We concede that our findings may be an underestimation of long-term PDD rates in the wider community as the present study only involved patients who attended our research clinics. While home visits were carried out for some participants in the Australian cohort, such visits were restricted to individuals enrolled into the brain donation programme. As such, there is likely an underrepresentation of long-term PD patients residing in nursing homes, thereby leading to an underestimation of the frequency of dementia in these patients. In addition, participants in the present study generally represent highly motivated and more highly educated individuals who therefore represent a form of “self-selection bias”.

One of the other limitations of this study relates to the retrospective nature of the study, which did not allow factors potentially affecting the outcome results to be accounted for (e.g. genetic information, the presence of other non-motor symptoms). Several genetic influences have been implicated in cognitive function in PD and it should be appreciated that the impact of specific gene mutations on cognition can be variable. While certain gene mutations (e.g. *PARKIN* mutations) are associated with a more favourable disease course in PD, other gene mutations including glucocerebrosidase (GBA) mutations^52–54^ and the presence of the MAPT H1/H1 genotype have been found to be associated a more rapid course of cognitive decline in PD patients and a higher risk of developing PDD.^55,56^ As genetic testing was not undertaken in this study, it is impossible to know the possible impact of such effects. However, none of the patients included were under the age of 30 years old at the time of their diagnosis, where one might expect more clear-cut genetic influences. Additional information on family history from the Australian cohort showed that only 4 of the 18 patients had a family history of PD, and 3 of these did not develop dementia. Genetic screening and longitudinal evaluation of gene mutations carriers would therefore be an important future direction for the investigation of genetic contributions to the development of dementia in long-term PD. Such investigation may also provide fundamental insights into the biophysical processes that underlie the disease pathogenesis in PD.^57^ It will also clearly be of value to have continuous data collected at different time points to investigate the evolution of motor and cognitive symptoms in long-term PD patients.

Another limitation of this study relates to the employment of the Level 1 criteria to classify PDD. Although it is suggested that the utilisation of additional neuropsychological tests may increase characterisation of PDD,^58^ the administration of a more comprehensive battery of neuropsychological tests may not always be practical or available due to the long administration time and increased possibility of fatigue, particularly in this population. On a related note, as participants’ performances in processing speed were not measured and accounted for within the context of the MDS Level 1 PDD criteria, consideration must be given when interpreting results from the lexical fluency item of the MMSE and MoCA, which although typically considered a measure of executive functioning, could be influenced by bradyphrenia. We also recognise the methodological issue of employing different measures to characterise PDD (i.e. MoCA in the Australian cohort versus MMSE in the UK cohort), which might have affected the characterisation of PDD cases in the UK and Australian cohort (i.e. 1 PDD case in the UK cohort versus 6 PDD cases in the Australian cohort). While both scales were recommended as useful tools to identify cognitively impaired patients with PD according to the MDS criteria for PDD^4,58^ and the MMSE has long been used as the primary method of defining PDD, MoCA has been suggested to be more sensitive than MMSE in characterising PDD cases. More specifically, MMSE may be less sensitive to more subtle cognitive dysfunctions in well-educated samples,^59–62^ and does not encompass items assessing executive functioning—which represents one of the cognitive domains commonly affected in PD.^63–65^ Furthermore, some of the items on the MMSE may not be directly comparable to that of MoCA (e.g. the drawing of intersecting pentagon on the MMSE versus that of a cube on the MoCA), and that MMSE is generally considered as less challenging than the MoCA^66^ (e.g. with shorter time span between immediate and delayed recall of words). Therefore, we acknowledge that the utilisation of the MMSE may potentially lead to an underestimation of PDD cases.

To date, there has been a paucity of data on long-term PD. Information on long-term clinical outcomes in PD is not only of great importance to the patient and their caregivers, but also for the purpose of identifying potential modifiable factors that could potentially halt or slow clinical progression. Findings from the present study suggest that the frequency of dementia in PD patients with 20 years or longer of disease duration may be lower than anticipated. While such findings cannot be directly compared with that of the Sydney Multicentre Study considering the inherent limitation of the retrospective, cross-sectional nature of the current study (e.g. the utilisation of different measures to characterise PDD across the Australian and UK cohort, not including all participants residing in nursing homes)—all of which could lead to an underestimation of PDD frequency, they suggested that the trajectory of cognitive decline in certain long-term PD patient can be different. It is well recognised that PD is a heterogeneous condition with many factors likely influencing cognitive decline and progression to dementia.^2,67,68^ Our findings highlight that the presence of more severe motor impairment is associated with the development of dementia in these patients. Conversely, educational attainment may serve as a protective factor against the development of dementia in long-term PD patients. Findings from the present study highlight the need to investigate factors that might affect the trajectory of cognitive decline in long-term PD patients, which may lead to the determination of potential modulating factors in the development of dementia in these patients.

## Methods

### Participants

The current study represents a multicentre, retrospective, cross-sectional study. An electronic database search was conducted for any PD patients with disease duration of ≥20 years across the Parkinson’s Disease Research Clinic at the Brain and Mind Centre, University of Sydney as part of an ongoing longitudinal research project. Participants of the study were also asked to consider donating their brains for diagnostic and research purposes. For participants enrolled into the brain donation programme, home visits were carried out in cases where they were unable to physically attend the clinic (e.g. due to difficulties with travelling or health reasons). The UK cohort originated from a patient database collected from Movement Disorders Specialist Centres across the UK as part of an ongoing non-motor international longitudinal study (NILS, UK Clinical Research Network (CRN) number 10084). Participants were required to have adequate proficiency in English for the assessment. Data from 2327 PD patients meeting the United Kingdom Parkinson’s Disease Society Brain Bank Criteria^69^ was pooled. This study was approved by each of the local ethics committees of the participating centres and the patients provided informed consent for the collection of demographic and clinical data.

### Clinical assessment

All participants underwent neurological assessment while on their usual medications. The neurological evaluation rated participants according to Hoehn and Yahr (H & Y) stages,^70^ and assessed them on the revised Movement Disorder Society Task Force Unified Parkinson’s Disease Rating Scale^71^ (MDS-UPDRS; for the Australian cohort) or the SCOPA-Motor Scale^72^ (SCOPA-M; for the UK cohort). Demographics and details of age at disease onset, disease duration, and medications were also recorded.

### Diagnosis of probable PDD

A diagnosis of probable PDD was made according to the Movement Disorder Society (MDS) Level 1 criteria.^4,58^ Probable PDD was diagnosed when all the following five criteria were satisfied:

1. *Diagnosis of PD according to United Kingdom Parkinson’s Disease Society Brain Bank Criteria*.^69^
2. *The development of PD prior to the onset of dementia:* This information was gathered by clinicians involved in assessing participants.
3. *PD associated with a decreased global cognitive efficiency:* This was defined by a cut of score of ≤25 on the Mini Mental Status Examination (MMSE).^73^
4. *Cognitive deficiency severe enough to impair daily life:* The foundation of a PDD diagnosis is that the cognitive decline contributes to functional impairments most commonly observed in the activities of daily living (ADLs). In the UK clinics, this was defined by the Clinical Impression of Severity Index for Parkinson’s Disease (CISI-PD).^74^ The cognitive status scale of this tool assesses the impact of cognitive decline on ADLs. The scale is scored 0–6, with 4 or higher suggesting that help is needed for ADLs, including help with basic daily activities. As such, any patient scoring 4 or higher met this criterion. In the Australian clinic, item 1.1 of the MDS-UPDRS^71^ was used. The scale is scored between 0 and 4 with a score of 2 or higher suggesting help is needed for ADLs, even if only minimal. As such, any patient scoring 2 or higher met this criterion.
5. *Impairments observed in more than one cognitive domain:* Impairments observed in the following cognitive domains was determined by performance on individual items in the MMSE in the UK clinics, and by performance on individual items in the Montreal Cognitive Assessment (MoCA)^75^ in the Australian clinic as individual item scores of the MMSE were not available.
  i. Attention: Both “Serial 7’s” or “Months Reversed” tests were available for patients in the UK clinics. Two incorrect responses on the former, and omission of two or more months, or incorrect sequencing or failure to complete the test within 90 s for the latter, were scored as impaired. “Serial 7’s” was available in the Australian clinic.
  ii. Executive functioning: Lexical Fluency, but not clock drawing was available for all patients, and any patient who scored less than 12 words in a minute was defined as impaired.
  iii. Visuo-constructive ability: Correct drawing of the MMSE Pentagons was employed to determine visuo-constructive ability in the UK clinics. With regard to the Australian clinic, the Cube copying component of the MoCA was used.
  iv. Memory impairment: The three-word recall of the MMSE was used for all patients in the UK clinics and any word missing was characterised as impaired. In the Australian clinic, delayed recall of the MoCA was used, where two words missing was recorded as impaired, given that five, rather than three, words were to be remembered.

### Motor phenotype

The current study compared tremor and non-tremor symptoms with the motor phenotype score based on an approach used previously.^67,68^ The score was obtained by dividing a patients’ tremor score by their non-tremor score. Aligning with the approach used in previous research, only items with the higher lateralised score (left or right) were selected. The tremor score represented the severity of subjective tremor and objective tremor at rest and during movements, whereas the non-tremor score assessed speech, facial expression, swallowing, ability to turn in bed, postural stability, walking and posture, rigidity, and global spontaneity of movement.

In the Australian cohort, the tremor score consisted of the mean of items 23, 54, or 55 (depending on the highest lateralised score), and 59 on the MDS-UPDRS, whereas the non-tremor score was derived from the mean of items 28–44 (only items with the highest lateralised score) of the MDS-UPDRS. With regard to the UK cohort, the tremor score consisted of item 1 (based on the highest lateralised score) on the SCOPA-M, and the non-tremor score consisted of the mean of items 3–5 (based on the highest lateralised score on each item) on the SCOPA-M. A tremor/non-tremor ratio of <1 indicates the dominance of non-tremor features (NTD), and a score of >1 indicates the presence of TD features.

### Statistical analysis

The Shapiro–Wilk test was performed to determine normal distribution. Comparison of demographic and clinical variables between the different groups was assessed using either an analysis of variance (ANOVA) or the Mann–Whitney *U* test (depending on whether the variable met parametric assumptions), except gender, for which the Chi-square (*χ*^2^) test was used. Subgroup analyses comparing the prevalence of PDD across patients with age of disease onset of ≥50 and ≤50 years, as well as age ≥70 years versus ≤70 years were also performed with the *χ*^2^ test to assess whether the disease onset age group and the chronological age group may be a factor in the recorded outcomes. All analyses employed an alpha level of *p* < 0.05 and were two-tailed.

## Supplementary information

Supplementary Informantion

## Data Availability

The data that support the findings of this study are available from the corresponding author upon reasonable request.
